# Supplementary material for: Genetic Dissection of Light-Regulated Adventitious Root Induction in Arabidopsis thaliana Hypocotyls
Source: Int J Mol Sci. 2022 May 10;23(10):5301. doi: 10.3390/ijms23105301 (PMC9140560; doi:10.3390/ijms23105301)
Supplement: Supplementary file 1 [file ijms-23-05301-s001.zip › ijms-1688256-supplementary.pdf]

Genetic dissection of light-regulated adventitious root induction in Arabidopsis hypocotyls

Yinwei Zeng [1], Sebastien Schotte[1], Hoang Khai Trinh[1,2], Inge Verstraeten[1], Jing Li [1], Ellen Van de Velde[1], Steffen Vanneste[1,3,4,‡], Danny Geelen[1‡]

Supplementary Materials:

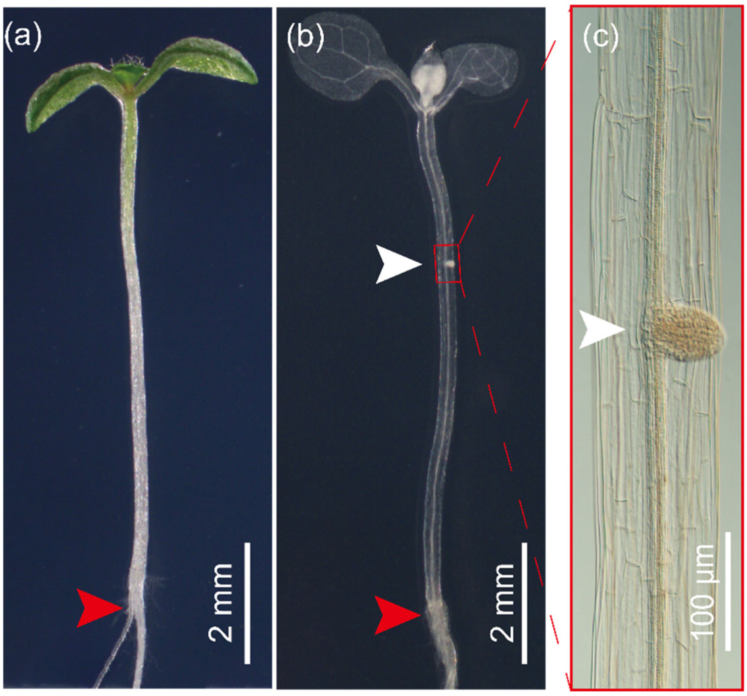

Figure S1. Arabidopsis hypocotyl clearing showing an adventitious root primordium. (a) Phenotype of Col-0 grown in the light for 4 days after 3 days etiolation; (b) Cleared hypocotyl; (c) detailed image of the ARP indicated in (b). White arrowheads indicate ARI site from hypocotyl, the red arrowhead points to the hypocotyl root junction.

Table S1. List of PCR primers.

Name	Locus tag	Primer sequences (5′ - 3′)
pif1-1_F	At2g20180	AAGGAAGGAGGAGGAATAGGC
pif1-1_R		CATGAATTTCTCGAGGCTGAG
pif3-7_F	At1g09530	AGAAGCAATTTGGTCACCATGCTC
pif 3-7_R		TGCATACAAATAGTCGATCGTATG
pif 4-2_F	At2g43010	AATTCATCATCGGGGATTAGG
pif 4-2_R		TCGTCGTTTAATAAACACGGC
pif 5-3 _F	At3g59060	CCGACTGTTATAACCGAGGATCT
pif 5-3_R		AACCGAGAAGGTTTTGGAGATAG
LBb1.3		ATT TTG CCG ATT TCG GAA C
